# Fostering implementation of knowledge into health practice: study protocol for the validation and redevelopment of the Knowledge Uptake and Utilization Tool

**DOI:** 10.1186/s12961-019-0503-5

**Published:** 2019-12-27

**Authors:** Sneha Shankar, Kelly Skinner, Melody E. Morton Ninomiya, Jasmin Bhawra

**Affiliations:** 1 Measurement, Evaluation and Research Methodology program, Department of Educational and Counselling Psychology, and Special Education, University of British Columbia, Vancouver, BC V6T 1Z4 Canada; 2 School of Public Health and Health Systems, University of Waterloo, Waterloo, ON N2L 3G1 Canada; 3 Department of Health Sciences, Wilfrid Laurier University, Waterloo, ON N2C 3C5 Canada; 4 Institute for Mental Health Policy Research, Centre for Addiction and Mental Health, London, ON N6G 4X8 Canada

**Keywords:** validation, knowledge uptake and utilization, integrated knowledge translation, knowledge translation, implementation, knowledge transfer and exchange, tools, unified validity theory

## Abstract

**Background:**

Measurement of what knowledge is taken-up and how that information is used to inform practice and policies can provide an understanding about the effectiveness of knowledge uptake and utilization processes. In 2007, the Knowledge Uptake and Utilization Tool (KUUT) was developed to evaluate the implementation of knowledge into practice. The KUUT has been used by numerous large health organizations despite limited validity evidence and a narrow understanding about how the tool is used in practice and interpreted by users. As such, the overall purpose of this protocol is to redevelop the KUUT and gather validity evidence to examine and support its use in various health-related organizations. This protocol paper outlines a validation and redevelopment procedure for the KUUT using the unitary view of validity.

**Methods:**

The protocol outlined in this article proceeds through four phases, starting with redeveloping the tool, then evaluating validity evidence based on: test content, response processes and internal structure. The initial phase gathers information to redevelop the tool, and evaluates item content and response format. The second phase evaluates response process validity evidence by examining how a variety of users interact with the tool. In the third phase, the tool will be pilot tested with knowledge users and, in the final phase, psychometric properties of the tool will be examined and a final scoring structure will be determined. A knowledge translation plan described herein outlines where the final tool will be housed and how the information about the tool will be disseminated.

**Discussion:**

This protocol outlines a procedure to gather different sources of validity evidence for the KUUT. By addressing limitations in the original KUUT, such as complexities with scoring, a redeveloped KUUT supporting validity evidence will enhance the ability of health-related organizations to effectively use this tool for its intended purpose.

## Background

Research in health settings aims to both improve knowledge and translate this knowledge beyond the individual to group or systemic levels. Measuring what knowledge is taken-up and how that information is used to inform practice and policies can provide an understanding about the effectiveness of knowledge uptake and utilization (KUU) processes. However, large gaps exist between the knowledge that is revealed through research and the knowledge that is used in practice. To address this gap, Skinner [1] developed the Knowledge Uptake and Utilization Tool (KUUT). Over a decade since its initial development, the KUUT has been used and adapted by a variety of large national level health organizations such as the Public Health Agency of Canada, Health Canada and the Canadian Partnership Against Cancer. Although there are no other broad measures that evaluate KUU that are known to experts in the field [2, 3], the KUUT has weak validity evidence and limitations with its current use (e.g. complexities with scoring). Furthermore, while the process of knowledge exchange between producers of knowledge as well as users of knowledge is dominant in healthcare policy [4] and has flourished there, the KUUT has also become relevant to other health-related organizations. As such, a more robust, redeveloped KUUT with stronger validity evidence to support its use is needed to confidently measure KUU and improve the ease of use. The purpose of this protocol paper is to outline the processes that will be undertaken to obtain validity evidence for an updated and redeveloped KUUT.

As a concept, KUU refers to the implementation of knowledge into practice within the broad field of knowledge transfer and exchange (KTE). How KTE is defined and operationalized varies greatly depending on many factors such as the disciplinary field, commitment to community, industry, government, level of stakeholder engagement and funding priorities as well as the background and training of researchers [5–7]. As noted by Graham et al., it has been well recognized and accepted that, in the last decade, a key aspect of KTE includes the integration of knowledge users during the research process to improve the “*relevance, applicability and impact of results*” ([8], p. 2). The idea of integrated knowledge translation promotes interaction and collaboration between researchers (or other producers of knowledge) and knowledge users in the KTE process. Subsequently, the concept of KUU involves an integrated KTE approach to communicate and collaborate with potential knowledge users, as well as the exchange and use of the knowledge by, and between, stakeholders (e.g. researchers, organizations) and knowledge users (i.e. general public, policy-makers) in both health and health-related organizations [4, 9]. Operationalization of KUU involves using research- or organizational-generated knowledge, which are often originally communicated in written form and then applying this knowledge into practice, policies or programme development [10–12].

Although KUU includes the process of KTE, it focuses more heavily on how knowledge users integrate knowledge products into their practice. Knowledge products include the ways knowledge is customized before it is spread to target audiences [11, 13], and in this study includes documents (e.g. reports, briefing notes, journal articles), data, websites, workshops, training and other activities used to share information with knowledge users. Effective dissemination of knowledge products (e.g. organizational reports) improves the chances that they are being used by policy, practice and programme decision-makers to generate larger and faster societal impact [4, 8]. Although it can be difficult to measure improvements or increases in the KTE process, such as through integrated knowledge translation or the uptake and utilization of knowledge products, there are increasingly more requests to apply and evaluate KTE efforts — and these components are what the KUUT aims to measure.

The importance of both identifying and measuring KUU has grown over the years. In the last decade, anticipated KTE goals, activities and rationale have been increasingly requested by funding agencies. It is rare, however, for funders, organizations or researchers to publish how the KTE efforts were taken up, utilized and resulted in change [14]. For example, lead funders for health research in Canada are increasingly funding more projects that are ‘partnered’, meaning that researchers must enter into a project with identified partners that will contribute, benefit from and/or use knowledge generated from a project [15, 16]. Studies reviewing KTE-related strategies focus on the impact of specific methods and very few evaluate the impact of KTE products or processes [17–20]. Furthermore, in a literature review of KTE practices and outcomes, no tools were identified to evaluate the uptake of knowledge products and the KUUT aims to fill this gap [21].

Given increased priority for research that demonstrates how findings will improve outcomes for individuals, communities or target populations, there is a need and appetite for a KUU tool with sufficient validity evidence to support its use [2, 3]. More specifically, validity information is needed to ascertain how the tool evaluates the concept of KUU. Validity is defined as “*the degree to which evidence and theory support the interpretations of test scores for proposed uses of tests*” ([22], p. 11). Investigating validity means evaluating how a tool is interpreted and the claims that are made about the tool [22, 23]. Validity information will also help to understand how the tool operates in different environments with different stakeholders. Currently, the existing validity evidence for the KUUT provides some preliminary information about test content by comparing the degree of association of knowledge use and KUUT outcomes, and uses qualitative interviews with a public health unit and the Level of Use scale [24, 25]. This evaluation found that there was 93% agreement between the KUUT and qualitative assessment [25]. As originally developed by Skinner [1], the KUUT is primarily informed by theory, including Knott and Wildavsky’s [26] Seven Standards of Utilization. In addition, two extra stages were included in the original 2007 KUUT [1] — awareness and effort. These stages were added by Skinner because 'awareness' of the knowledge product (or knowledge process) is necessary to take up and utilize the knowledge, and the knowledge user making an 'effort' to use that knowledge is a component of utilization. Altogether, the KUUT considers nine categories that are necessary for effective KUU (Table 1). However, the existing validity evidence does not establish how well the theoretical evidence is represented in the measure or how well the tool represents the concept of KUU. The protocol presented in this paper considers the gaps in validity evidence for the KUUT using current measurement perspectives from the unified validity theory [22, 27].

**Table 1:** Stages/Standard of Knowledge Utilization^a^

Stage	Category	Description
	Awareness	Awareness of the information^b^
1	Reception	Receiving information/information is within reach
2	Cognition	Read, digest and understand information
3	Discussion	Altering frames of reference to the new information
4	Reference	Information influences action/adoption of information
	Effort	Effort to favour information over others
5	Adoption	Influences, outcomes and results
6	Implementation	Adopted information becomes practice
7	Impact	Tangible benefits of information

^a^Stages 1–7 summarized from Knott and Wildavsky [26] with the categories ‘Awareness’ and ‘Effort’ added by Skinner [1]

^b^The term ‘information’ could be substituted by any of the following: document, evaluation, initiative, innovation, intervention, knowledge, practice, policy, product, programme, project, research, etc. (Skinner [1])

The overall purpose of this study is to redevelop the KUUT and gather validity evidence for its use in health and health-related organizations. This paper outlines a validation protocol for the redevelopment and testing of the KUUT using the unitary view of validity. In particular, several sources of validity evidence will be explored, which include test content, response processes and internal structure. These sources of validity evidence will (1) provide information about the relevance and representativeness of items towards the KUU (test content), (2) evaluate how respondents both use and interpret the tool (response processes), and (3) provide preliminary psychometric evidence to confirm its factor structure and reliability (internal structure). These sources of evidence build an integrative judgment of how well the tool evaluates KUU, and helps to support the inferences that are made about the test score [28, 29]. In outlining the protocol for this investigation, this study uses integrated knowledge translation approaches and offers an example for how the unified validity theory can be applied, as application of this modern validity approach continues to be underutilized [22, 30, 31]. Altogether, the research protocol outlined here and subsequent evidence will contribute towards how well the tool evaluates the concept of KUU. Evaluating and strengthening validity evidence for the KUUT will aid implementation of this concept by knowledge users. It will also help organizations to determine the most effective ways of disseminating and using knowledge internally, and prompt knowledge users to consider how they might use new evidence that would be of interest to many organizations.

## Methods

**Fig. 1:**
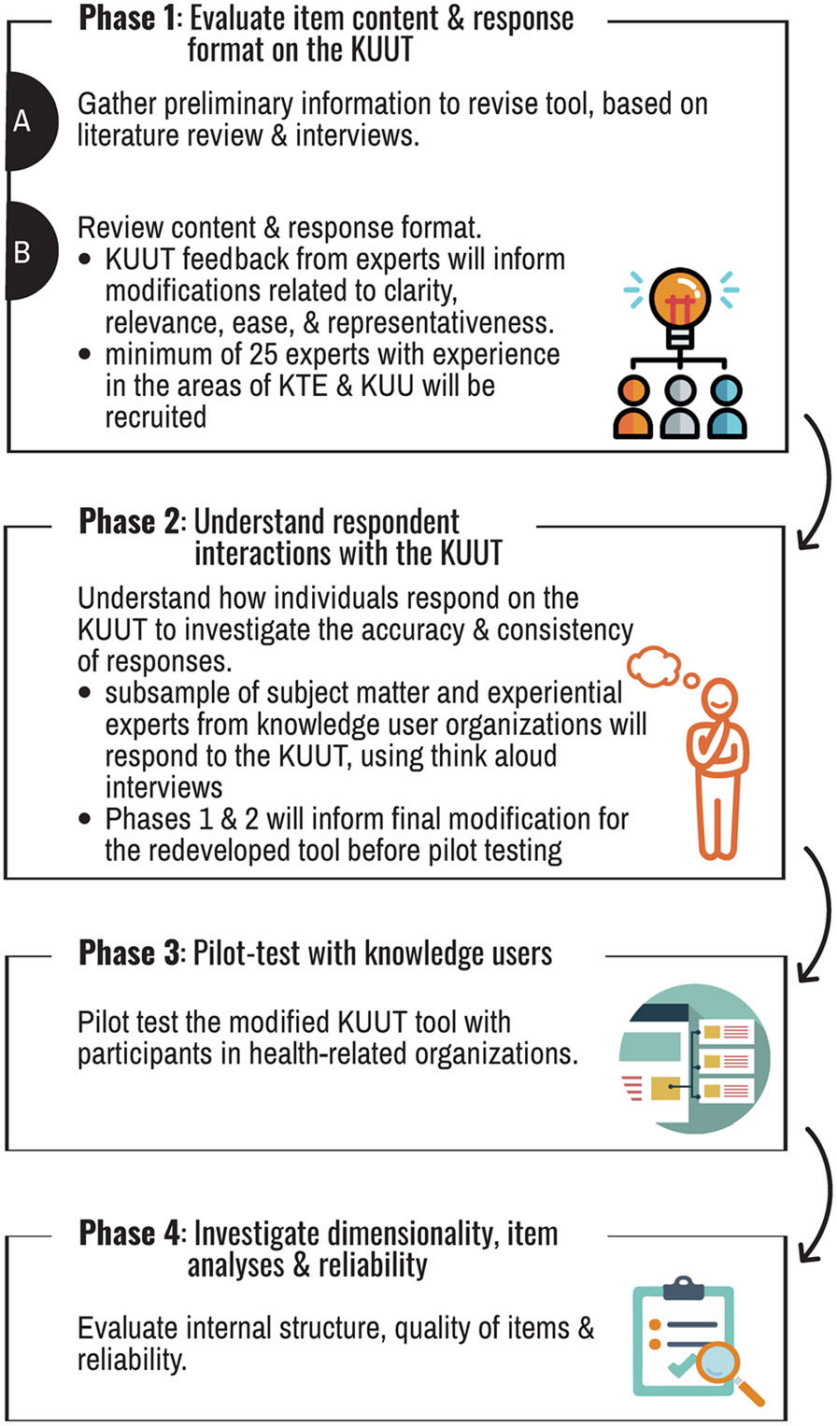
Redeveloping the KUUT and gathering validity evidence

Scale development and validation practices that employ the unified validity theory [22, 27] will inform the redevelopment and validation processes of the KUUT at all stages.Within the unified view, validity is centred around the construct or the unobservable phenomenon that one aims to measure [22, 32, 29]. The research protocol outlined herein includes four phases that will redevelop the KUUT and gather different sources of validity information. The protocol starts by revising the original KUUT, then proceeds to gather information and evaluate the following sources of validity: test content, response processes and internal structure. Each phase is outlined in detail below, including a description of the process as well as participants and subsequent analysis (Fig. 1). A knowledge translation plan for disseminating the redeveloped KUUT is also included.

### Phase 1: evaluate item content and response format on the KUUT

This initial phase will revise the KUUT using information from a literature review and interview feedback from users of the KUUT. Next, the tool content will be tested by asking experts to provide feedback about items, response format and instructions.

#### Part 1a – gather preliminary information to revise the tool

Since the original development of the KUUT by Skinner [1], both informal and formal feedback has been received about its content and response format. This initial phase consists of gathering preliminary information from a literature review, as well as interview feedback about the KUUT from KUUT users (ORE#31771). A scoping review by the research team has begun to explore the various tools that are used to evaluate KTE processes. Ongoing interviews with KUUT users are providing insight about the types of organizations, projects, knowledge products or processes that the KUUT is being used for as well as how the KUUT is being adapted to fit different contexts. Using the aforementioned information as well as guidelines in tool development and validation practices [22, 33], the original KUUT is undergoing revisions by two researchers (SS and KS). Items are being re-worded, added, or removed and the response scale refined. Revision of items includes consideration of their relevance to theory and how each item relates to the concept of KUU. Overall, the scale is being simplified and shortened to facilitate the ease of use, as well as a greater ability to investigate psychometric properties.

#### Part 1b – review content and response format

In this phase, information about the redeveloped KUUT will be received from experts and any further modifications will be made regarding its clarity, relevance, ease and representativeness. Specifically, information about the test content, response format and instructions will be evaluated.

#### Participants and analysis

Gathering validity evidence based on test content will begin by assembling subject matter experts who are knowledgeable in the fields of KTE, KUU and evaluation, as well as experiential experts who have practical experience with KTE and KUU. Subject matter and experiential experts will be invited to examine the content of the KUUT and judge the relevance and representativeness of items included in the redeveloped tool. We anticipate the group of subject matter and experiential experts to include a minimum of 25 people who will be recruited from several health-related organizations.

Both subject matter and experiential experts will judge the content of the KUUT using an online or in-person survey. Respondents will provide feedback on the shortened KUUT about the following: (1) clarity of administration, (2) clarity of instructions, (3) relevance of items to KUU and (4) ease of response format. In addition, respondents will provide narrative feedback for each of the items in the KUUT and judge the appropriateness of each item towards the overall scale. A content validity index [34] will be used to indicate the proportion of experts (both subject matter and experiential) who endorse each of the items. Any items that have a content validity index of less than 0.80 will be marked for review [35].

Qualitative information about the content will be gathered by suggestions from subject matter and experiential experts about items that need to be modified from their narrative feedback. To evaluate the narrative feedback, content analysis [36] will be employed to organize descriptions, followed by themes to summarize feedback at the item and overall test level. This information, along with information gathered in Phase 2, will be used to inform further modifications to the KUUT.

### Phase 2: understand respondent interactions with the KUUT

Response processes provide validity evidence by explaining how respondents interact with a measure and relating this information to how a test is used and interpreted. Response processes are an aspect of validity that examine the process models involved in a measurement task and include aspects such as cognitive processes [32]. In order to understand how individuals respond the way they do on the KUUT, this aspect of the research study will investigate the accuracy and consistency of responses to the KUUT using a combination of think-aloud interviews and verbal probing. Using the process of a think-aloud interview, also termed concurrent verbalization, respondents will be asked to think out loud as they are completing the measure and the interviewer will also probe about specific information, such as why an item may reflect a particular theoretic stage or ways the item can be reworded [37]. During the think-aloud interview, respondents will be asked to describe the things that come to mind as they are responding to items on the measure. Interviewers will probe for more information to prompt respondents to explain what they are thinking or saying to themselves.

#### Participants and analysis

A small subsample of both subject matter and experiential experts from each of the knowledge user organizations in Phase 1 will be asked to respond to the KUUT while thinking aloud. Using think aloud interviews will enable researchers to examine what, if any, differences exist between various knowledge users and an opportunity to draw specific considerations for sex and gender of users, as well as ensuring diversity of the sample. Respondents will be asked to think out loud as they are completing the measure and the interviewer will also probe about specific information, such as why an item may reflect a particular theoretic stage or ways the item can be reworded [37]. The information gathered here will help to justify interpretations of this tool by providing evidence for how and why users respond the way they do [28]. Altogether, the information obtained from Phases 1 and 2 will inform final modifications before pilot testing.

### Phase 3: pilot testing with knowledge users

The modified KUUT will be pilot tested with participants in various health-related organizations.

#### Participants and analysis

Pilot testing will involve real world application of the redeveloped KUUT with various knowledge user organizations. Each knowledge user organization has individuals who are recipients of disseminated knowledge products; these individuals are henceforth referred to as ‘secondary knowledge users’. During pilot testing, knowledge users will introduce a new knowledge product to their secondary knowledge users and, approximately 2–3 months after the new knowledge product is introduced, the secondary knowledge users will complete the redeveloped KUUT. As a period of time is required for secondary knowledge users to take up and utilize a disseminated knowledge product, this is why the KUUT will be administered 2–3 months after dissemination. Opportunities for knowledge user products include the following:

- Secondary knowledge users at the 36 public health units will receive various knowledge products from Public Health Ontario;
- Successful applicants of the Sunnybrook Health Sciences Centre seed grant programme, who are required to disseminate the knowledge products that they produce;
- The Canadian Observatory on Homelessness would like to measure the impact of the evidence they are building through their Making the Shift and A Way Home Canada projects — more specifically, through 12 demonstration projects being conducted in 10 communities in the provinces of Alberta and Ontario, they are testing intervention models aiming to support young people as they exit homelessness or the risk for homelessness; and
- Homewood Research Institute has a recovery monitoring system that synthesizes data from patients into a dashboard that is being developed to provide outcome reports for clinicians.

A month after the KUUT is first used, re-test information will be gathered by the same participants. The aim is to have a total sample size of at least 300 secondary knowledge users to pilot test the redeveloped tool and to ensure a strong sample size for subsequent psychometric testing in the next phase.

### Phase 4: investigate dimensionality, item analyses and reliability

Using the data gathered in Phase 3, the redeveloped KUUT will be evaluated for internal structure to examine dimensionality. In addition, the quality of items and reliability will be evaluated.

#### Analysis

Factor analysis will be used to examine the dimensionality of the tool and confirm the scoring structure. Using an exploratory factor analysis, the scale will be evaluated to explore dimensionality. In addition, item analyses will be conducted to analyse the quality of items and determine the final selection of items (e.g. inter-item correlations, item-total and corrected item-total correlations, alpha-if-item-deleted, and factor loadings). Internal consistency and test–retest reliability estimates will also be obtained.

### Knowledge translation plan

This study also includes its own knowledge translation plan strategy that will, through the process of gathering validity information for this tool (also acting as a form of integrated knowledge translation) and through dissemination of the final KUUT, engage KTE and evaluation hubs such as the Knowledge Translation Canada, the Knowledge Translation Program at St. Michael’s Hospital, Institute for Knowledge Mobilization and Canadian Evaluation Society. In addition, organizations such as the Public Health Agency of Canada, Health Canada, the National Collaborating Center for Public Policy, and Public Health Ontario will be involved in the knowledge translation plan. We will also network with other organizations that might have an interest in measuring KUU. The redeveloped KUUT will be housed in an open access format on Dr Skinner’s website [38] and will be disseminated through webinars open to any organization. During the end of the KTE phase when we disseminate the final KUUT through presentations and webinars, we will also evaluate the usefulness of the KUUT [39] through an online survey with new users and determine whether it is more useful for some users than others (e.g. for non-profit vs. government organizations; research vs. evaluation utilization; large vs. small organizations, etc.).

## Discussion

The value of research, for community-based and government organizations alike, is based on the usefulness of the knowledge produced [40, 41]. Furthermore, health-based organizations rely heavily on knowledge products, such as evidence-based research, to develop or improve policies and programmes. Government-funded projects that aim to address priority issues such as poverty, homelessness or mental health frequently support intervention research or implementation science projects with a strong evaluation component that measures change (e.g. Homelessness Hub, Local Poverty Reduction Fund of the Ontario Trillium Foundation, etc.). The capacity for organizations to evaluate how knowledge products and processes have been taken up greatly varies [42] and the KUUT serves to fill this need.

While this protocol details how validity evidence will be gathered for the KUUT, it is important to understand subsequent conclusions that can be made about this tool once this validity evidence is obtained. Within the unified view, validity is no longer considered from a dichotomous perspective, where validity evidence determines whether a tool is ‘valid’ or ‘not valid’, or deems a tool as conclusively ‘validated’ [22]. The shift in conceptions of validity from a fixed or static property requires consideration that validity is an evolving concept that changes with time and use, in various settings [32]. As such, validity information is contextualized and dependent on the use of the tool. Gathering validity information and the processes involved in redevelopment of a tool will highlight the strength of evidence to support the use of the tool in different health-related organizations. Although application of the unified validity framework remains largely underutilized [28, 29], this validity approach encourages an understanding of how the tools are interpreted by users and is currently endorsed by the Standards for Educational and Psychological Testing [22]. Furthermore, as recommended in studies that investigate validation practices, articulating validity approaches are initial steps that support the foundation of a strong validity argument [22, 28]. Applying this framework also recognizes the consequences and possible negative implications of testing, which are important but often overlooked considerations [43]. Overall, the information gathered in this protocol will provide information about how the KUUT is used and understood in practice, thereby strengthening the validity evidence for this tool. Whether the validity evidence is sufficient to warrant use in settings that are different from those tested here is uncertain; however, we aim to test this tool with a wide audience to meet the needs of, and support its use with a number of interested health-related organizations.

## Conclusion

This validation protocol will provide evidence for the KUUT to evaluate how well knowledge dissemination, uptake and utilization, or transfer of knowledge products are used by knowledge users and stakeholders, including diverse groups of women, men and gender-diverse individuals. The KUUT may also be used to hold researchers and other knowledge producers accountable, help organizations determine the most effective ways of disseminating and using knowledge internally, and prompt knowledge users to consider how else they might use new evidence that would be of interest to many organizations. A redeveloped tool with validity evidence to support it allows relevant organizations to confidently use the tool to measure KUU and make appropriate inferences from the measure.

## Data Availability

Not applicable.

## Abbreviations

KTE: knowledge transfer and exchange
KUU: knowledge uptake and utilization
KUUT: Knowledge Uptake and Utilization Tool
